# Filling Teeth

**Published:** 1858-09

**Authors:** J. Taft


### DENTAL REGISTER OF THE WEST.

VOL. XII.—SEPTEMBER, 1858.—NO. I.

### Original Essays and Communications.

### FILLING TEETH.

BY J. TAFT.

#### BLOCK FILLING.

Another, and in some respects a far preferable method, is filling with blocks. Some of the advantages of this method over that just described, are the following: The filling can be introduced far more rapidly, and the lamina, or leaves of foil occupy a more perfect position in the cavity, and consequently the structure of the filling is better. The form of the cavity should be much the same as that for any other method of filling; there should be some retaining point so situated that the first block, or blocks, can be fixed firmly in place, so that there will be no liability of it becoming loosened during the subsequent part of the process. It is important to have such an arrangement, otherwise it would be necessary to employ an instrument in the left hand to retain the first blocks in situation until enough were introduced to bind the whole by pressure upon two opposite points in the cavity.

*Forming Blocks.*—For forming the blocks, use any number of foil that may be desired, usually No. 4 or 6, and either lay four to six sheets together, or fold a single sheet into that number of thicknesses ; then cut off, of the sheets thus prepared, strips about one-third to one-fourth wider than the depth of the cavity to be filled, these are then rolled on a small three or four sided broach—the three is better ; this instrument should be very small, no larger than is necessary for strength. The sides should be perfectly smooth, and the angles sharp and smooth ; ordinarily it should not taper, or at least very slightly, if at all. For forming the conical blocks, some prefer the tapered broaches, but they can be as well made on the parallel sided instruments. Taking the strip between the thumb and index finger, it is rolled on the broach equally, till the block or cylinder is large enough, when the strip is broken off. The size of the principal part of the blocks should correspond with the size of the cavity to be filled. Different sizes and forms will be required in almost all cases. Relatively large cylinders may be employed for the principal part of the filling. If the walls of the cavity are parallel, almost all the blocks may be truly cylindrical ; but if there is an under dipping of one or more of the walls, the blocks adjusted to that particular part should be cone-shaped, corresponding to that under-dipping. A number of graduated small cone-shaped blocks, of different degrees of density, will be required for completing each filling ; as the aperture becomes smaller, less blocks will be needed. The cone-shaped blocks are formed, by gradually running the strip back from the point of the instrument as it is wound on ; greater or less taper can be given to it, as the strip is run less or more rapidly back from the point. The density of the block can be regulated by the firmness with which the strip is held between the thumb and finger ; upon these it is well to have a fine silk, or India-rubber covering to protect the gold from the perspiration of the hand. There are other methods of forming blocks. They may be made square, by making a great number of folds—fifteen to thirty—and from this cutting strips as before directed, and then from these heavy strips, cutting off the blocks of the desired size, these will then be flat or nearly square. In one respect they are objectionable, the edges when they have been cut off are rendered dense by the action of the shears, so that they do not possess the uniform density or consistence of the rolled blocks, and it is impossible to adopt them as perfectly to the walls of the cavity, or to one another.

Another method of forming blocks, employed by Dr. Blakesley, of Utica, N. Y., is to roll a sheet of No. 5 foil into a rope, and cut off from it blocks, one-fourth larger than the depth of the cavity to be filled. These are liable to the same objection as those immediately preceding, by the shears hardening them, when they are cut off. They are subject to an additional objection, the folds of foil are not as regular as by either of the other methods. The cavity formed, and the blocks prepared, the next step is their introduction.

*Introducing the Block.*—For placing the gold into the cavity the plugging pliers will be required. The points should be curved, so as to make the most perfect approach to the cavity. The points of these, if properly formed, may be used for condensing the blocks.

All things being ready, with suitable napkins and guards for the protection of the cavity against the encroachment of moisture from the saliva and breath, the fingers of the left hand should be placed firmly on the napkin, and also to hold away the soft parts. If there is a very acute angle, a small block should be first introduced with the pliers into the proper position, one end upon the bottom of the cavity, and the other protruding from the orifice, pressure should then be made upon it to consolidate it and force it into its position against the wall of the cavity. This may be done with the pliers, or probably better with an instrument formed for the purpose. The part of the instrument brought to bear upon the gold, should be roughened either longitudinally or transverse, so that a proper surface may be left for the reception of the succeeding portions, the largest blocks are then introduced and consolidated successively as described. The end of each left protruding till the gold is all introduced, each portion, as it is introduced, should be perfectly condensed. The gold should be filled in faster at the sides of the cavity than in the center, and in this way filling the gold around upon the walls until it meets at a point in the cavity opposite the place of beginning; thus the gold is adapted to all the walls of the cavity before it is entirely filled, the last portions being introduced somewhere near the center of the filling. As the cavity diminishes by the introduction of the gold, the small and more dense blocks will be required; these should be forced in and condensed, by forcing an instrument down against the side of the cone. Some operators terminate the filling against the wall of the cavity, forcing down the blocks and compressing, as above, till it is full. By this method there is danger of fracturing the tooth, breaking down the wall of the cavity, when the filling is terminated. Another method is to fill up the cavity principally with blocks, and the last part of the filling is put in, in the strip filled in from the bottom to the orifice.

The objection to this method is, that unless adhesive foil is employed, the portion put in, of strip, is liable to be displaced, and in this way the whole filling becomes destroyed.

Another method of arranging this kind of filling, particularly when the bottom of the cavity is irregular, is to make a large, flat pellet, and press firmly into the bottom, and set the blocks upon this for a foundation. By this method there is a more perfect adaptation of the gold to the bottom of the cavity, by placing the ends of the blocks down upon the bottom of the cavity; there may be a failure in this respect, without great care.

After the gold is all introduced, a small pointed plugger must be passed all over the surface, to consolidate the protruding portions of gold and form a surface to the filling. The protruding portion should be sufficient to make the surface perfectly flush with the border of the cavity, for a depression here is fatal to a perfect finish. After the surface is condensed with the fine and large points, it may be rubbed down with an instrument serrated upon the side, and after this the coarse file, and then the fine, etc.

Dr. Badger describes a method of filling a small cavity on the posterior approximal portion of a second molar, the third molar gone. The cavity was formed with a bur drill. A cylinder was then formed in the usual manner, and forced through a series of holes in a draw-plate, down to the size of the bur with which the cavity was formed. The block was then quite dense, the cavity was then dried, and the block forced into it, which it exactly fitted, and protruded a little from the orifice. This block was pierced in the center with a sharp instrument, and a small dense roll forced into it; this was then all condensed and finished in the usual manner.

*Pellets.*—Round pellets, made by rolling fragments or pieces of foil between the thumb and fingers, are used by some operators, and with them they profess to make as good filling as by any other method. They are made of various sizes, and packed into the cavity with sharp or serrated pointed instruments. The pieces may thus be very solidly worked together, and a good filling made, provided the pellets are not too large; they should be small enough, that the point or points may work through them into the proceeding portions. Unless this is observed, a good filling can not be made with pellets. Some operators use pellets and crystal gold together. This may do very well, if the adhesive property of the gold is employed; but either one alone would answer in this condition. There can not be as much gold put in by this mode as in the blocks well adjusted.

*Adhesive Foil.*—By this we understand that condition of gold in which its leaves unite very readily and very firmly together. This property of cohesion is possessed, in the greatest degree, by properly manufactured foil, immediately after annealing. This does not impart any new property to the gold, but removes obstacles to the manifestation of a principle possessed by all gold, under favorable circumstances. It is now about four years since this property was first employed in gold foil for filling teeth. To Dr. R. Arthur, of Baltimore, is due the credit of first directing the attention of the profession to this subject. He not only did this, but he entered most fully into the details of the manipulations, instruments, etc., pertaining to this mode of operating. Almost all recently prepared gold foil possesses this property to a greater or less degree, there are methods of preparing it, however, by which it possesses it most fully; all recently annealed foil is adhesive.

If the foil is adhesive when we wish to use it, nothing farther is required in the way of preparation. But if it is not adhesive—as almost all foil is not, especially if it has been exposed to the influence of the atmosphere—it will require to be made so by some process; there are two, either of which may be employed, which will perfectly accomplish the object.

The one most frequently employed, is heating the gold, either in the sheet, the roll, or fragments, over the flame of a spirit lamp, almost or quite to a red heat; if in the sheet, it should be laid upon a piece of wire gauze and passed over the flame of the lamp for a moment or two; if in the roll, it may be taken in the center with fine pliers and passed rapidly through the flame; if in small fragments or pellets, placed upon a piece of charcoal and a light flame thrown upon them with the blow-pipe. The other method is that adopted by Dr. Coates, of Virginia. It consists of a little platinum pan, large enough to hold a sheet of foil, without folding; into the pan is put from one to two gills of rain water, add to this about forty drops of sulphuric acid, place it over the flame of a spirit lamp till it boils a few moments. The acid removes all foreign substances from the surface of the gold. Remove it from the boiling liquid, and in a moment it is dry and ready for use, and will be most perfectly adhesive.

Having the gold in its most perfect condition, there are different methods of using it, the cavity should be formed in general, about as for the other methods of filling, except that to retain the first piece, there should be two or three small pits or holes made for retaining points, in the most available position.

The first portion of gold should be a little pellet; this forced into these retaining points, serve as a foundation for the retaining portion of the filling. Dr. Arthur's method is, then, to tear off fragments from the sheet and pass it into the cavity without folding up, and condense it with a fine serrated pointed instrument, so that it not only unites by cohesion but it is worked into the surface of the preceding portion of gold; and in this manner, portion after portion is introduced and condensed, until the cavity is full. The filling may be commenced in any part of the cavity that is most convenient; in many, as in crown cavities of the molars, at the bottom and filled to the orifice. In putting in the gold, it should, during its introduction, be kept fuller about the walls of the cavity than in the center; by this means the adaptation will be most perfect to the walls, and there will be no liability of clogging up the center. The gold may thus be built up to any desired extent if the filling is kept dry—moisture is fatal to the adhesion of gold. Others use the adhesive gold in a different manner from that described above. To Dr. Blakesley of Utica, belongs the honor of first detailing the following plan. The sheet of gold may be folded or not at the pleasure of the operator, and then each sheet cut into two to six strips, and these formed into a loose roll between the thumb and fingers; it may now be passed rapidly through the flame of a spirit lamp to remove any foreign substance that may be upon it; it is now cut into little blocks or pellets, these must be of various sizes; the size of the pieces may be regulated by the size of the roll and the amount that is cut off. The gold thus prepared, and the cavity ready, the filling is to be introduced; for this about three sizes of serrated pointed instruments are required, those having fine points are preferable ; for the size of these points, Dr. Blakesley remarks, "they will just enter, respectively, No. 22, 24 and 26 of the wire gauge." A larger than either of these is desirable for many cases. As before, the filling may be commenced at the bottom of the cavity or at one side if desirable, with a large pellet, sufficiently large to be set firmly into the retaining points. Then take up the small pellets or blocks upon the point of the plugging instrument, and place them exactly in the desired point and consolidate them perfectly, building up next to the wall all around higher than the center, using the smaller pieces to fill up the small corners and interstices; for filling these small spaces the smaller points will be required. The gold is then packed in till the cavity is full, then finish as usual. Another method is to tear off fragments from the sheet, and roll these up into round pellets and fill with these, with the same instruments and upon the same principle as above described. With these it is difficult to make a perfect filling—the gold is liable to clog in the cavity and fail in adaptation.

Adhesive gold must be consolidated as it is introduced, for after a cavity is full, though it may be but partially consolidated, it will be very difficult to do anything farther in that direction—far less can be done with non-adhesive foil.

